# Role overload, pain and physical dysfunction in early rheumatoid or undifferentiated inflammatory arthritis in Canada

**DOI:** 10.1186/1751-0759-6-13

**Published:** 2012-05-03

**Authors:** Sally Sabry Mustafa, Karl Julian Looper, Phyllis Zelkowitz, Margaret Purden, Murray Baron

**Affiliations:** 1 Department of Psychiatry, McGill University, Montreal, Quebec, Canada; 2 Department of Nursing, McGill University, Montreal, Quebec, Canada; 3 Department of Rheumatology, McGill University, Montreal, Quebec, Canada; 4 Department of Psychiatry, McGill UniversityJewish General Hospital 3755 Chemin de la Côte-Ste-CatherineMontreal, Quebec H3T 1E2, Canada

**Keywords:** Arthritis, Role overload, Physical functioning, Pain

## Abstract

**Background:**

Inflammatory arthritis impairs participation in societal roles. Role overload arises when the demands by a given role set exceed the resources; time and energy, to carry out the required tasks. The present study examines the association between role overload and disease outcomes in early inflammatory arthritis (EIA).

**Methods:**

Patients (*n =* 104) of 7.61 months mean duration of inflammatory arthritis completed self-report questionnaires on sociodemographics, disease characteristics and role overload. Pain was assessed using the Short Form McGill Pain Questionnaire (MPQ) and physical functioning was measured with the Medical Outcomes Study Short Form 36 (SF-36) physical functioning score. Role overload was measured by the Role Overload Scale. Patients indicated the number of social roles they occupied from a total of the three typical roles; marital, parental and paid work.

**Results:**

Participants’ mean age was 56 years and 70.2% were female. Role overload was not correlated to the number of social roles, however, it was positively associated with pain (*p* = 0.004) and negatively associated with physical functioning (*p* = 0.001). On multivariate analysis, role overload was negatively associated with physical functioning after controlling for the relevant sociodemographic variables.

**Conclusion:**

This study identifies a possible reciprocal relationship between role overload and physical functioning in patients with EIA.

## Background

Arthritis is a prevalent chronic illness in Canada. In 2008, 4.2 million (16%) Canadians 15 years or older had arthritis. Arthritis was the second most prevalent chronic condition among women and the third among men, preceded only by back pain and hypertension. Prevalence of arthritis increased with age, yet, 58% of patients were younger than 65 years. Arthritis was almost twice as prevalent in women as in men, regardless of age [1].

Disability is difficulty functioning due to structural/functional bodily impairment, activity limitation (difficulty executing tasks such as climbing stairs) or participation restriction (difficulty getting involved in life situations) [2]. In 2001, 3.75% of Canadians reported disability participating in societal roles such as paid work and family due to arthritis [1]. Arthritis prevalence was higher among the separated or divorced compared to those living with spouse. This could stem from the strain arthritis puts on family dynamics and/or economics with the ultimate consequence of separation. Regarding paid employment, arthritis patients had to reduce working hours, change jobs, undergo workplace modifications, work from home, or ultimately quit [3-5].

The large societal costs of arthritis can be minimized through early treatment. There exists a crucial “window of opportunity” when disease course can be altered. Arthritis medications include analgesics, non-steroidal anti-inflammatory drugs, corticosteroids, disease modifying anti-rheumatic drugs such as methotrexate and the newest biologicals such as the tumor necrosis factor alpha inhibitor adalimumab. Biologicals and disease modifying anti-rheumatic drugs effectively prevent joint damage, limit disease progression and even achieve full remission thus enhancing the overall quality of life and enabling patients fulfill their social roles such as maintaining employment [6,7].

Arthritis refers to more than a hundred joint and surrounding tissue rheumatic disorder. Inflammatory arthritis is joint inflammation due to immune disruption. It includes rheumatoid arthritis (chronic destructive synovitis) and seronegative arthritis. Early inflammatory arthritis (EIA) has no agreed upon definition but is used to describe any inflammatory arthritis condition of certain maximum duration, ranging from 16 weeks to 36 months [8]. Undifferentiated arthritis is one which does not fulfill any specific set of disease classification criteria. Little is known about the incidence and prevalence of EIA. Early treatment prevents the transition of undifferentiated arthritis to classified rheumatoid arthritis or early rheumatoid arthritis to the fully blown one [8]. In Canada, early detection of arthritis is attempted through a self-administered arthritis screening tool used by patients suffering early symptoms of musculoskeletal disorders [9].

A social role is a position held in a social institution. Examples of social institutions are the family and the economic system. Position holders in these institutions, also known as role occupants or actors, are expected to behave according to certain standards. Role occupancy describes the number of positions people hold across various social institutions. Examples of roles highly salient and hence co-occupied by most people are paid work, personal relationships such as marriage, parenting and friendships, caregiving, household as well as leisure activities such as travel or sports. Occupying multiple social roles is likely to cause role stress, because of the limited nature of human energy, according to the role strain hypothesis. Role overload is a form of role stress that reflects, mainly, time constraints. Role balance, on the other hand, refers to the smart distribution of resources across diverse roles giving rise to well being and sense of accomplishment rather than role strain [10-13].

Participation in social roles continues to be highly valued by people with arthritis despite their suffering [14]. However, this is not an easy task. Occupying multiple social roles was associated with higher levels of role stress; role conflict and role overload, less role balance and hence less psychological well being in women with rheumatoid arthritis [15]. Multiple social roles also increased the fatigue related negative emotions experienced by younger women with rheumatoid arthritis [16]. Achieving role balance between inflammatory/osteoarthritis, paid work and personal life roles proved stressful. Role overload was evident as arthritis, paid work or both compromised the time available for personal life activities eventually leading to role loss; leisure activities being the first to give up [17]. Individuals with osteoarthritis were low to moderately satisfied with their performance and the time they spent in roles they considered highly salient [18]. Arthritis patients rated higher and hence were more protective of their involvement in the labor force and social relationships as compared to physical leisure or household activities [17,18].

Disease factors can also play a role in arthritis. Pain was perceived by inflammatory and osteoarthritis patients as an aggravating factor for role conflict [17]. Greater pain was associated with less satisfaction with role performance and time spent on roles in osteoarthritis [18]. Pain limited activities such as getting in and out of a car which in turn restricted participation in social and leisure tasks [19].

The present study centers on the concept of role overload [20]. The role overload construct was developed in the social sciences and was researched in relation to global health measures but rarely to specific disease processes. Role overload evaluates the pressure from the viewpoint that the time available is too short for all roles to be performed effectively. It thus detects how overwhelmed, rushed and uncomfortable people might feel towards their life responsibilities. Moreover, the role overload construct captures the overall/cumulative effect of all roles without having to individually assess them.

Although the body of evidence relating role difficulties to arthritis is growing, sufficiently missing is the possible association between disease variables and perceptions of role overload. In an attempt to fill this void, the present study examined the relationship between pain, physical functioning and role overload. We postulated that worse disease outcomes such as more intense pain and compromised physical functioning will be associated with higher role overload in patients with EIA.

Past literature indicating role overload in arthritis patients occupying multiple social roles [15,16] encouraged us to examine the same in our sample. Our approach was to reduce to the three social roles basic to most arthritis patients as supported by the literature [17,18]; namely paid employment and the social relationships spouse and parent.

The majority of participation literature in arthritis focused on longstanding illness populations [15]. In the present study we stress the early stage of the illness when the major disruption to social roles occurs, adjusting the roles and accommodating the illness are most needed and interventions proved most effective [21].

The current study investigated both biological (pain and physical functioning) and psychosocial (role occupancy and role overload) factors in arthritis. Its aim was to contribute towards securing full participation and role balance to individuals burdened with arthritis.

## Methods

### Participants

Two hundred and fifty seven patients were enrolled in the McGill Early Arthritis Registry (McEAR) in the period between March 2006 and May 2009. Referrals to the McEAR came from 21 rheumatologists working in Montreal, Canada. The participating rheumatologists, each of whom works in a private office or outpatient hospital clinic, were asked to recruit all new EIA patients who fit the inclusion criteria. Those agreeing to participate were then referred to the registry. Patients were included in the McEAR if they had newly diagnosed inflammatory arthritis, defined as one or more inflamed joints of a minimum duration of six weeks to a maximum duration of 18 months. Patients were 18 years or older, spoke English or French, and agreed to periodic physical and laboratory examinations as well as to completing questionnaires assessing demographics, disability, pain and psychosocial factors related to their illness. Exclusion criteria included clinical evidence of remote joint damage suggestive of a previous episode of rheumatoid arthritis, any rheumatic diagnosis other than rheumatoid arthritis or undifferentiated inflammatory arthritis, severe functional limitation from a disease other than arthritis, and any disorder that compromised the ability to give informed consent. These patients were seen by the study nurses who traveled to meet them at the office of the referring rheumatologist with whom the clinical care remained. This made it geographically easier for the patient who lived anywhere on, or just off, the island of Montreal. There were two nurses working on the registry. Each was trained to perform a complete tender and swollen joint count.

All subjects enrolled in the McEAR Registry were invited to participate in this study. A total of 104 patients (40.5% of the total McEAR participants) recruited from the McEAR between March 2006 and May 2009 agreed to participate. A trained interviewer arranged to see the patient at home within ten days of the registry visit, obtained consent for the study and assisted the patients in filling out the study specific questionnaires. Patients received $25 for each meeting with the study interviewer.

All patients in the McEAR signed an informed consent and the study was approved by the research ethics committee of McGill University and the Jewish General Hospital.

### Measures

#### Illness outcome measures

##### *Physical functioning*

Physical functioning was measured with the Medical Outcomes Study Short Form 36 (SF-36), which has been frequently used in medical populations and has good psychometric properties [22]. The SF-36 is an instrument used to measure health-related quality of life. It consists of eight domains: physical functioning, social functioning, role limitations (physical problem), role limitations (emotional problem), mental health, vitality, bodily pain, and general health perceptions. Scores range from 0 (worst) to 100 (best). The 8 scales can be combined into two summary measures: the Physical Component Score, which provides overall estimate of physical health, and the Mental Component Score, which estimates overall mental health. In this study we used the physical functioning score, which measures physical functioning in an exclusive way. An example question is “Does your health now limit you in bathing or dressing yourself?” We avoided the Physical Component Score because it takes into consideration other components such as bodily pain. This reduced potential overlap between our measure of physical functioning and the other measures used in this study such as the McGill pain Questionnaire (MPQ).

##### Pain

Pain was assessed using the Short Form MPQ [23,24]. It contains 11 items related to the sensory dimension of pain and four related to the affective dimension. Each descriptor is ranked on a four-point intensity scale (0–3; none to severe), and total scores range from zero (no pain) to 45 (severe pain). The MPQ has been extensively used and has sound psychometric properties. The total pain score was used in the current study.

#### Social roles measures

##### Number of social roles

In our assessment, we counted the number of social roles our participants occupied from a total of three roles: spouse, parent and/or paid worker.

#### Dependent measure

##### Role overload

Role overload was measured using the Role Overload Scale (ROS) [13]. ROS is a self report measure consisting of 13 items scored on a five-point Likert scale ranging from one to five in correspondence to “strongly disagree” to “strongly agree”. This scale measures the three general aspects of role overload namely too many demands, not enough time, and not enough energy to complete role tasks satisfactorily, for example: “I never have enough time to do everything that is expected of me”. Item-total correlations ranged from .501 to .797, and the Cronbach’s alpha for the role overload scale is .88 [13]. The scale provides a single total score ranging from 13 to 65, the higher score indicating higher role overload.

#### Statistical analysis

Means, standard deviations and frequencies were calculated for all study variables. Pearson correlations were used to calculate the associations among continuous study variables. Independent samples *t* tests were used to detect differences in role overload (ROS) among dichotomous study variables. Multiple linear regressions were used to demonstrate the association between illness measures and role overload while controlling for relevant sociodemographic variables. All statistical tests were two sided, and a *p* value of 0.05 or less was considered significant. Analyses were performed using SPSS, version 17 (SPSS, Chicago, IL, USA).

## Results

Our sample consisted of 104 patients with a mean age of 56 years. The majority (70.2%) was female living with spouse and 27.9% had children under 18 years old living at home. Almost half (49.0%) of the participants were engaged in paid work. The average yearly household income was distributed as follows: 38.5% earned less than $60 000, 41.3% earned $60 000 or more and 17.3% refused to answer. Regarding education level, 45.2% of the participants had high school education or less and 53.8% had college, university or post-graduate education. Participants in this study had a mean duration of illness of 7.61 months. Of our patients, 79.8% were diagnosed with undifferentiated and 20.2% with rheumatoid arthritis.

The overall McEAR population had an average age of 56.18 (14.69) years, 67.7% were female, 62.2% lived with spouse and 26.9% had children living at home. Average disease duration was 6.75 (6.65) months. These descriptive characteristics were not significantly different from the group that participated in this study.

The mean number of social roles occupied by our patients was 1.45 roles. Of our patients, 16 (15.4%) occupied all three measured roles; namely, spouse, parent and paid worker, 34 (32.7%) occupied two of the three roles, 35 (33.7%) occupied one role and 19 (18.3%) did not occupy any of the specified social roles.

Means, standard deviations of the means and frequencies of all study variables are described in Table 1.

**Table 1:** Descriptive statistics for study variables

**Variable**	**N**	**Mean (SD) or %**
**Age (years)**	104	56.09 (14.39)
**Female**	104	70.2%
**Living with spouse**	104	70.2%
**Participants has children under 18 living with them**	104	27.9%
**Participant has paid work**	104	49.0%
**Yearly income:**	101	
**< $60 000**		38.5%
**≥ $60 000**		41.3%
**Education level:**	103	
**High school or less ** **College or more**		45.2% 53.8%
**Disease duration (months)**	104	7.61 (3.90)
**MPQ total pain**	93	8.60(9.86)
**SF-36 physical functioning**	94	54.96(27.13)
**Number of social roles**	104	1.45(0.96)
**Role Overload Scale (ROS)**	104	33.76(12.42)

The level of role overload (ROS) was not significantly correlated to any of the demographic variables measured, namely, age, sex, living with spouse, having children under 18 years old in the same household, paid employment, yearly household income or education level. Similarly, role overload did not significantly correlate with the number of social roles or disease duration. However, ROS did significantly correlate with MPQ total pain. The relation was in the positive direction. This means that the more intense is the pain the higher the role overload (or vice versa). ROS also correlated with SF-36 physical functioning (Table 2). This time the correlation was in the negative direction implying greater overload with lower physical functioning abilities.

**Table 2:** Pearson correlations (*r*) among Role Overload (ROS) and study variables

	**Statistics**	**Number of social roles**	**MPQ total pain**	**SF-36 physical functioning**
**1. Role Overload**	*r*	.137	.297	-.333
	*p*	.169	.004	.001
	*n*	103	92	93

Table 3 shows the multiple linear regression model for the dependent variable role overload, controlling for relevant sociodemographic variables. The overall model and the independent variable indicating physical functioning were significant, adjusted R^2^ = 0.114, F(df = 8,82) = 2.453, *p* = 0.020. However, the independent variables of age, sex, living with spouse, having children under 18 living at home, having paid work as well as the disease variables disease duration and pain were not significant.

**Table 3:** Multiple linear regression model for Role Overload (ROS)

**Dependent Variable: ROS**						
					**95 % Confidence Interval for B**	
**Independent variables**	**B**	**SE B**	***t***	***p***	**Lower Bound**	**Upper Bound**
**Constant**	45.569	10.007	4.554	.000	25.662	65.476
**Age**	-.117	.119	-.983	.328	-.354	.120
**Sex**	.727	2.865	.254	.800	−4.972	6.425
**Living with spouse**	2.077	2.821	.736	.464	−3.536	7.689
**Having children under 18 living with them**	3.132	3.608	.868	.388	−4.046	10.310
**Having paid work**	−1.071	2.828	-.379	.706	−6.698	4.556
**Disease duration**	-.390	.317	−1.230	.222	−1.020	.240
**MPQ total pain**	.179	.143	1.251	.215	-.105	.463
**SF-36 physical functioning**	-.125	.054	−2.309	.023	-.232	-.017

Adjusted *R*^2^ = 0.114.

## Discussion

According to the results of the present investigation, the hypothesis that, in EIA, role overload and the disease variables pain and physical dysfunction are interrelated is confirmed. Univariate correlation analysis showed that, poorer physical functioning and severer pain are associated with higher role overload. Multivariate regression analysis revealed that worse physical functioning and role overload stay related no matter what the potential confounding variables. In other words, the present study found that patients do feel they do not have enough time and/or energy to get their social roles fulfilled. Pain and physical dysfunction were related to this sensed role overload, however, in which way is yet to be determined. It could be that pain and physical dysfunction are making role overload worse or it could well be that they are getting worse by role overload. Role overload was previously seen to correlate to illness severity in women with rheumatoid arthritis [15]. However, the study by Coty & Wallston (2008) differed from ours in that the number of social roles occupied by patients was positively related to role stress; role conflict and role overload. That is, rheumatoid arthritis women who reported occupying more roles also reported higher levels of role stress. In our study, however, engaging in multiple social roles was not significantly associated to role overload. The discrepancy could be due to the larger numbers of roles included in the other study (nine roles; wife, mother, paid worker, unpaid worker at home, caregiver of spouse or parent, student, volunteer, friend, or grandparent), a mean of 5.8 roles. This is in contrast to the three typical social roles selected for our study; namely spouse, parent and/or paid worker, and a mean of 1.45 roles carried out by our patients. Other explanations of the discrepancy in results could relate to gender where all their patients were women while ours were 30% men with the consequent differences in sex roles, and the chronic duration of illness in the population they studied; mean disease duration of six years as compared to 7.61 months in ours.

Our results are also in agreement with Gignac *et al* (2012) who found role overload to be evident in individuals with inflammatory or osteoarthritis [17]. Similar to our findings, the authors reported pain, fatigue, symptom unpredictability and disability to interfere with work and personal roles. Ultimately, many patients were forced out of some of their roles, especially socializing, leisure and household activities. The authors warned that role overload may eventually lead to role loss, an alarming consequence. The study by Gignac *et al*. was qualitative while we measured our associations in a quantitative way.

Gignac *et al* (2012) found arthritis patients to be highly protective of their paid employment even at the expense of their own health [17]. In addition, Gignac *et al* (2008) found that social relationships are identified by osteoarthritis patients as most important [18]. Patients were relatively more satisfied with their performance and the time they spent in those social relations than physical leisure, travel, and vacation. The lack of association between role overload and number of roles in our study might thus be a reflection of the esteem our patients had for these three important roles (spouse, parent, paid worker) that compensated for or masked the strain they might have been producing.

### Limitations

One of the limitations of the present study is the relatively small sample size which limits the complexity of analyses and the generalisability of the findings. It could be that we lacked the power to detect significant associations among some study variables such as the number of social roles and role overload. Nevertheless, we were able to demonstrate significant relationships between role overload and disease variables. A second limitation is the cross sectional nature of the study which does not allow the direction of effects to be determined. We are therefore not sure whether it is the pain and physical dysfunction that need to be addressed in order for role overload to be lowered in patients with EIA or is it the other way round. Longitudinal studies are clearly needed to improve our understanding of how disease progression may affect participation in societal roles and the time constraints involved. A third limitation is the use of self-report measures including the dependent measure. However, the instruments assessing disease variables in this study; namely, SF36 and MPQ are widely used in the medical literature and have well established psychometric properties. The ROS, however, was rarely used in arthritis. We thus lack the information about the validity (content and construct) and reliability of the ROS in people with arthritis. Future research is definitely needed to address this gap. Finally, the present sample excluded patients with diagnoses other than early rheumatoid or undifferentiated inflammatory arthritis. Future studies should either include other diagnoses included under the definition of inflammatory arthritis such as spondyloarthritis and systemic lupus erythematosus, or be specific to only one type of inflammatory arthritis, for example, rheumatoid arthritis (the most common) to obtain comparable data.

## Conclusion

The present study highlights the burden that impaired physical functioning, pain and distress around timely fulfillment of social role demands can pose on patients in the early months of their journey with arthritis. This implies interrelatedness between patients’ physical health and participation in valued life activities which calls for a multifaceted approach regarding the care for patients with arthritis. Not only are the physical symptoms of arthritis to be managed, but also the emotional distress attendant upon the pain and functional impairment associated with the disease. Rheumatologists could be invited to include social role participation issues in their global care of patients and/or refer to allied health care professionals for further assessment on an individualized basis. Gignac *et al.* (2012) actually found that increasing instrumental support helped role overloaded arthritis patients meet their role demands [17]. Our study’s emphasis on the very early stage of the disease points towards the necessity of addressing role overload from the onset of illness when approaching patients starting a struggle with inflammatory arthritis.

## McGill Early Arthritis Research Group

Michael Starr, MD^3^, Michel Gagné, MD^3^, Michael Stein, MD^3^, Harb Kang, MD^3^, Morton Kapusta, MD^3^, François Couture, MD^3^, Mary-Ann Fitzcharles, MD^3^, Bruce Garfield, MD^3^, Henri A. Ménard, MD^3^, Laeora Berkson, MD^2^, Christian Pineau, MD^3^, Andrzej Gutkowski, MD^3^, Michel Zummer, MD^4^, Jean-Pierre Mathieu, MD^4^, Suzanne Mercille, MD^4^, Sophie Ligier, MD^4^, Jiri Krasny, MD^3^, Carole Bertrand, MD^4^, Sai Yan Yuen, MD^4^, Jan Schulz, MD^3^.

^2^Division of Rheumatology, Sir Mortimer B. Davis – Jewish General Hospital

^3^Division of Rheumatology, McGill University, Montreal, Quebec, Canada

^4^Service de rhumatologie, Hôpital Maisonneuve-Rosemont, Montreal, Quebec, Canada

## Competing interests

The authors declare that they have no competing interest.

## Authors’ contributions

All authors made substantive intellectual contributions to the study. KL, PZ and MP effected the conception and design, and acquisition of data. MB carried out measurement of disease (arthritis) outcome measures. SM performed the statistical analysis and interpretation of data and drafted the manuscript and took full responsibility for the article during submission and peer review. All authors revised the manuscript critically for important intellectual content and gave final approval of the version to be published.
